# The Importance of Vigilant Prescreening and Monitoring: Missed Adrenal Insufficiency in a Patient on Pembrolizumab

**DOI:** 10.1155/crom/8656099

**Published:** 2025-09-29

**Authors:** Juned Islam, Zakaria Rashid, Alex B. Munster, Peta Hughes, Sasa Badzek

**Affiliations:** ^1^Medical Oncology, Whittington Hospital, London, UK; ^2^Faculty of Medical Sciences, University College London, London, UK; ^3^General Medicine, Basildon University Hospital, Basildon, UK

**Keywords:** adrenal insufficiency, case report, immune-related adverse events, immunotherapy, pembrolizumab, immune checkpoint inhibitors

## Abstract

Immune checkpoint inhibitors (ICIs), such as pembrolizumab, have transformed cancer treatment by enhancing antitumour immunity. However, they are associated with immune-related adverse events (irAEs), including endocrinopathies like adrenal insufficiency. While existing literature extensively covers the risk of pembrolizumab-induced adrenal insufficiency, this case highlights the consequences of failing to act on an abnormal pretreatment cortisol result, leading to delayed diagnosis and clinical deterioration. We report the case of a 76-year-old male with metastatic squamous cell carcinoma (SCC) of the oesophagus undergoing treatment with pembrolizumab. A routine pretreatment blood test in mid-September revealed a cortisol level of 55 nmol/L (low), but this result was not flagged or acted upon. Three weeks later, the patient presented with worsening lethargy, vomiting and hyponatraemia. Follow-up testing confirmed persistently low cortisol levels, leading to a delayed diagnosis of adrenal insufficiency. Hydrocortisone replacement therapy was initiated, with subsequent symptom improvement. This case underscores the need for rigorous pretreatment hormone profiling, systematic review of laboratory test results and safeguarding mechanisms such as automated alerts for abnormal findings. Implementing structured endocrine monitoring in immunotherapy prescribing protocols could prevent similar occurrences and improve patient outcomes.

## 1. Introduction

Pembrolizumab, a checkpoint inhibitor, has shown significant promise in treating various cancers by stimulating the immune system to attack cancer cells via the PD-1/PD-L1 pathway [1]. However, like other immune checkpoint inhibitors (ICIs), pembrolizumab can result in immune-related adverse events (irAEs), which include endocrinopathies such as adrenal insufficiency [2]. This condition can be life-threatening if not promptly diagnosed and treated. Here, we present the case of a 76-year-old male with metastatic oesophageal cancer undergoing palliative pembrolizumab therapy. The patient was later diagnosed with adrenal insufficiency, which was not promptly addressed due to missed opportunities for early intervention. This case highlights the importance of vigilant monitoring and immediate action when abnormal cortisol levels are identified in patients receiving ICIs.

## 2. Case Presentation

**Figure 1:**
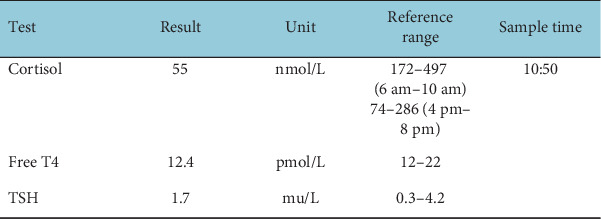
Pretreatment blood tests (mid-September 2024).

The patient's treatment history included receiving FOLFOX and pembrolizumab chemoimmunotherapy (without use of steroids throughout) between March and September 2024, before switching to 6-weekly maintenance pembrolizumab alone. The pretreatment bloods prior to Cycle 1 of maintenance pembrolizumab were taken on 17 September 2024. The cortisol result was alarmingly low at 55 nmol/L (Figure 1). Despite this, the result was not acted upon, and the pembrolizumab was administered without further investigation into the potential cause of the low cortisol levels.

**Figure 2:**
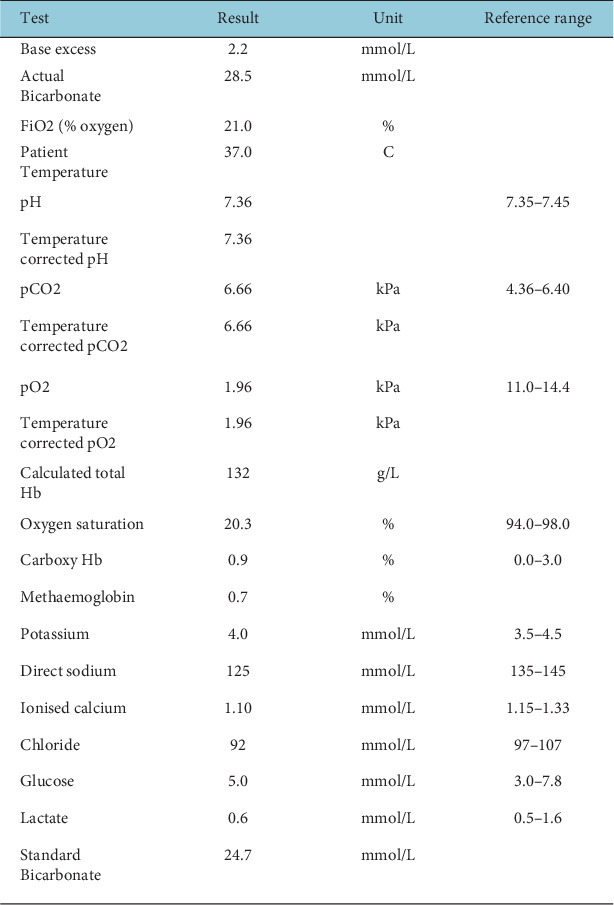
Emergency department admission blood tests (4 October 2024).

On 4 October 2024, the patient presented to the emergency department with fatigue and vomiting, with blood tests revealing a low sodium level of 125 mmol/L and a low cortisol level of 39 nmol/L (Figure 2). He was diagnosed with adrenal insufficiency, and oral hydrocortisone replacement therapy was initiated, which followed the 10/5/5 daily dosing regimen (10 mg at 8 AM, 5 mg at midday and 5 mg at 2 PM) [3, 4], resulting in symptomatic improvement. However, on 28 October 2024, prior to his second cycle of pembrolizumab, cortisol levels were again low at 35 nmol/L. Despite this, the patient went on to receive a further cycle of pembrolizumab. This compounded the previous error, as his adrenal insufficiency was still not appropriately corrected. On 6 December 2024, a treatment–response CT scan showed stable liver disease with improved lymph node size reduction. As a result, the patient proceeded to pembrolizumab Cycle 3 without reevaluating his cortisol levels despite the previously noted issues.

**Figure 3:**
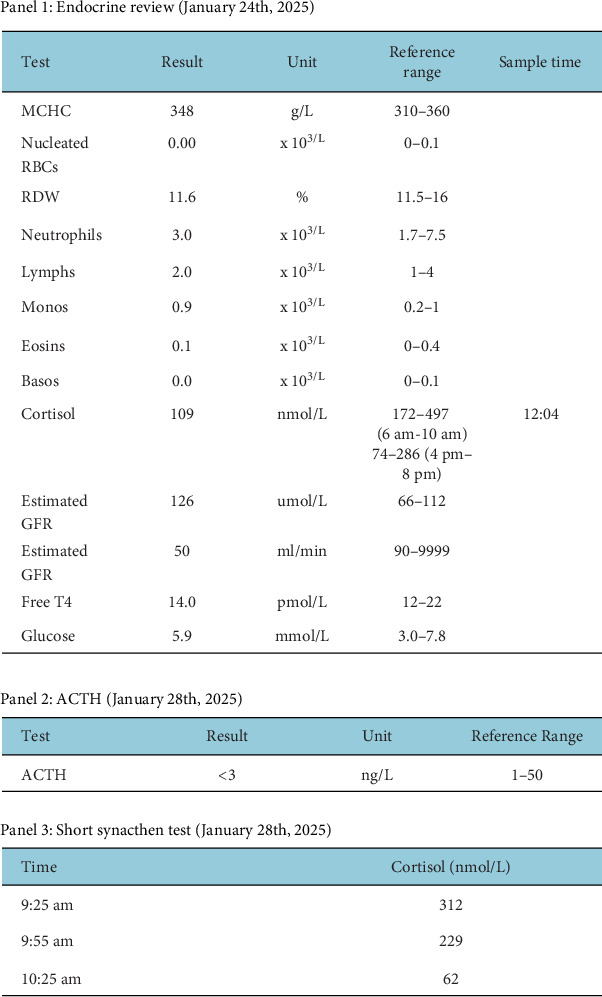
Follow-up endocrine blood test panel (24 and 28 January 2025).

On 4 January 2025, the patient reattended A&E with light-headedness. There was no evidence of postural hypotension, and blood testing for glucose and serum electrolytes returned normal values. A cortisol level of 400+ nmol/L was noted, and as such, the patient was reassured and discharged on his continued hydrocortisone therapy. A postdischarge review by endocrinology noted abnormal thyroid function tests (TFTs) from 9 December 2024, with a TSH level of 3.3 and a low free T4 level of 9.9, suggesting hypophysitis. On 24 January 2025, cortisol levels were measured at 109 nmol/L (Figure 3 – Panel 1), with interpretation difficult due to the suboptimal timing of the test; however, the patient proceeded with pembrolizumab Cycle 4. An ACTH level and short synacthen test (SST) were scheduled for 28 January 2025 to assess adrenal function further; this revealed an ACTH level < 3 (Figure 3 – Panel 2) and a drop in cortisol level from 312 to 229 to 62 from 9:25 AM to 9:55 AM to 10:25 AM, respectively, confirming secondary adrenal insufficiency (Figure 3 – Panel 3).

By 11 March 2025, the patient's cortisol had risen to 491 nmol/L before his fifth cycle of pembrolizumab, and a subsequent CT scan on 17 March 2025 showed a marginal reduction in liver lesions; thus, his treatment remains ongoing at the present time. Given the suspicion of immune-mediated hypophysitis, the patient is currently awaiting a confirmatory MRI of the pituitary and a full pituitary blood profile as of 14 April 2025.

## 3. Discussion and Recommendations

Our case supplements the body of similar cases of pembrolizumab-induced adrenal insufficiency that have been described in the literature, reinforcing the importance of early recognition and intervention. For instance, Alnahhas et al. reported a case of myxedema coma and adrenal insufficiency following pembrolizumab, underscoring the risk of multiple concurrent endocrinopathies with ICIs [5]. Likewise, Yamaguchi et al. described a case of pembrolizumab-associated adrenal insufficiency presenting with nonspecific symptoms such as fatigue and hyponatraemia, highlighting the diagnostic challenge [6]. These reports, alongside our case, demonstrate the spectrum of presentations and the need for systematic endocrine monitoring in patients treated with PD-1 inhibitors.

**Figure 4:**
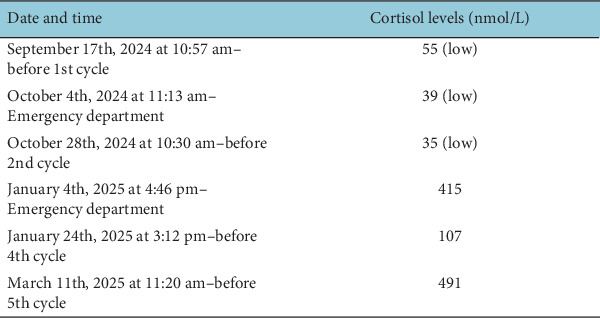
Table showing cortisol fluctuations over time.

This case highlights a preventable delay in diagnosing adrenal insufficiency due to a failure to act on an abnormal cortisol result. The primary issue was the missed opportunity for early intervention, as the patient's low cortisol levels were not flagged appropriately at multiple points (Figure 4). Cortisol is a critical marker for detecting adrenal insufficiency, yet its monitoring is often inconsistent in patients receiving ICIs [7]. The European Society for Medical Oncology (ESMO) Clinical Practice Guidelines for the management of toxicities from immunotherapy emphasise the importance of baseline and repeated endocrine evaluation, including thyroid hormone and cortisol levels, to identify preexisting conditions, establish a baseline and facilitate follow-up monitoring [8]. In this case, the patient's initial cortisol level of 55 nmol/L, although below the normal range, was not addressed appropriately. Both the European Society of Endocrinology (ESE) and National Institute for Health and Care Excellence (NICE) guidelines recommend that oral steroid therapy should be used in patients with secondary adrenal insufficiency, preferably a total dose of hydrocortisone of 15–25 mg given twice or thrice daily [3, 4]. By the time the patient presented with worsening symptoms, adrenal insufficiency had progressed significantly, leading to a delayed diagnosis and treatment. Several recommendations can help improve adherence to clinical guidelines and prevent missed opportunities for early detection and intervention.

Cortisol levels should be taken at standardised times for accurate interpretation [9]. As cortisol levels fluctuate diurnally, with peak values in the early morning (before 9 AM), testing outside these windows can lead to inaccurate results. In this case, cortisol samples were not consistently collected at the appropriate times, contributing to potential misinterpretation. To improve diagnostic accuracy, healthcare professionals should receive training on the importance of timed cortisol testing and adhere to best practices when monitoring adrenal function.

Cortisol results should be mandated as critical tests on clinical platforms such as ChemoCare and CareFlow Proforma, alongside other routine blood results. A structured template should include endocrine markers, such as cortisol and TFTs, as part of the monitoring for immunotherapy patients. To streamline this, a dropdown menu could be added to proformas to select the treatment type (chemotherapy, immunotherapy, targeted therapy or endocrine therapy). Selecting immunotherapy should automatically include an immunotherapy-specific template with cortisol levels. Including cortisol in routine immunotherapy monitoring would enable clinicians to more easily track potential adrenal insufficiency and take appropriate action [7]. Furthermore, a simple yet effective measure to ensure abnormal cortisol results are not missed would be to visually highlight them, for instance, in red, similar to other abnormal critical parameters.

A major challenge here was the delay in reporting cortisol results, hindering timely intervention. Policies should ensure cortisol results are reviewed before treatment [10]. This could involve earlier testing in the treatment cycle to allow review time before the next infusion. Alternatively, administration should only proceed once cortisol (and other hormone) levels are deemed within the normal range in patients receiving anticancer treatments, such as ICIs, that carry a higher risk of iatrogenic endocrinopathy [11].

AI-driven alert systems in electronic health records (EHRs) could enhance the detection of abnormal cortisol levels. Predictive analytics powered by machine learning could track cortisol trends and trigger alerts upon deviation [12]. Additionally, real-time smart alerts with escalation protocols should be integrated into EHR systems. Unlike standard flagging mechanisms, these alerts would trigger immediate notifications to primary clinicians. Such systems have been shown to enhance diagnostic accuracy and improve timely medical interventions [13]. Wearable devices combined with AI could further improve outcomes by continuously monitoring patient vitals and health metrics [14]. This real-time detection could help bridge the gap between blood test abnormalities and clinical action, ensuring timely interventions and better patient outcomes.

Managing immunotherapy-induced endocrinopathies requires a multidisciplinary approach. Oncology teams, endocrinologists and pharmacists must collaborate to monitor for adrenal insufficiency and other adverse events. Regular reviews of test results, alongside ongoing education for clinicians and patients, are essential for early detection and intervention [11].

## 4. Conclusion

This case demonstrates the critical importance of timely and accurate monitoring of adrenal function in patients receiving pembrolizumab therapy. The missed opportunity to act on abnormal cortisol levels resulted in a delayed diagnosis and treatment of adrenal insufficiency, which could have been avoided with proper clinical vigilance and adherence to established guidelines such as those from ESMO. Recommendations for improving clinical practice, including timely cortisol testing, standardised documentation, multidisciplinary collaboration and clear protocols for acting on abnormal results, are essential to enhance patient safety and ensure that similar mistakes do not occur in the future.

## Data Availability

The data that support the findings of this study are available on request from the corresponding author. The data are not publicly available due to privacy or ethical restrictions.
